# Adjuvant Chemotherapy for Stage II Colon Cancer

**DOI:** 10.3390/cancers12092584

**Published:** 2020-09-10

**Authors:** Sara Elena Rebuzzi, Guido Pesola, Valentino Martelli, Alberto Felice Sobrero

**Affiliations:** 1 Medical Oncology Unit 1, IRCCS Ospedale Policlinico San Martino of Genova, Largo Rosanna Benzi 10, 16132 Genova, Italy; saraelena89@hotmail.it (S.E.R.); guido.pesola@student.unisi.it (G.P.); martellivalentino91@gmail.com (V.M.); 2 Medical Oncology Unit, Department of Medicine, Surgery and Neurosciences, Azienda Ospedaliera Universitaria Senese, Viale Bracci, 53100 Siena, Italy

**Keywords:** stage II colon cancer, adjuvant chemotherapy, risk factor, survival benefit, algorithm

## Abstract

**Simple Summary:**

Stage II colon cancer is defined as an early stage of the tumor disease, without the involvement of lymph nodes or distant organs. In this group of patients, surgery alone is associated with high cure rate and the role of post-operative chemotherapy is still a matter of debate. In patients with tumor features associated with a high risk of recurrence, post-operative chemotherapy is recommended even if it has a small survival benefit. This clinical issue leads to the need for identifying patients who may benefit from post-operative chemotherapy based on their risk of recurrence. The purpose of this review is to highlight and discuss the uncertainties of the previous trials about the risk stratification, the weight of each prognostic factor and the therapeutic benefit of adjuvant chemotherapy in stage II colon cancer patients. Moreover, we summarize the data from previous studies in a decision algorithm that could help clinicians in clinical practice.

**Abstract:**

In stage II colon cancer management, surgery alone has shown a high cure rate (about 80%), and the role of adjuvant chemotherapy is still a matter of debate. Patients with high-risk features (T4, insufficient nodal sampling, grading, etc.) have a poorer prognosis and, usually, adjuvant chemotherapy is recommended. The purpose of the present study is to highlight and discuss what is still unclear and not completely defined from the previous trials regarding risk stratification and therapeutic benefit of adjuvant chemotherapy. With all the limitations of generalizing, we make the effort of trying to quantify the relative contribution of each prognostic factor and the benefit of adjuvant chemotherapy for stage II colon cancer. Finally, we propose a decision algorithm with the aim of summarizing the current evidence and translating it to clinical practice.

## 1. Introduction

Adjuvant chemotherapy is the standard of care for stage III colon cancer after surgical resection as it provides an absolute long-term survival benefit between 8.7% and 21.5%, depending upon the T-and N-related subclassification (Table 1) [1]. This survival gain does not hold up in stage II, where surgery alone has a high cure rate and the role of adjuvant chemotherapy with fluoropyrimidines has a small benefit (below 5%) [2]. In fact, different trials and meta-analyses have shown conflicting results regarding the size of the benefit of adjuvant therapy in stage II, if any at all. In particular, there is uncertainty over the gain with fluoropyrimidines in overall survival (OS) (0%, 3%, or 5%). This small benefit is due to the fact that most of the stage II patients have a very good long-term survival, leaving a relatively small risk to be reduced by adjuvant therapy [3].

This clinical issue leads to the need for risk stratification, with the purpose of identifying patients who may be candidates for chemotherapy based upon their high risk.

However, the proper definition of high and low risk is not completely clear in stage II; a cut-off of 20% is commonly used in clinical practice, taking into account different features such as T, N, grading, emergency surgery, etc. Unfortunately, we are not yet able to estimate precisely the risk of recurrence of the combination of these factors in every single patient.

Hence, open questions concern not only which patients with which high-risk features will benefit from adjuvant therapy but also what type of chemotherapy (monotherapy versus (vs) doublet therapy) is recommended and which survival end-point should be established in stage II-specific clinical trials, given the uncertain surrogacy of disease-free survival (DFS) for OS in this stage.

The purpose of the present study is to highlight and discuss what is still unclear and not defined from the previous trials, hoping that available big data could help to answer the still open questions regarding patient risk definition and stratification and therapeutic benefit. With all the limitations of generalizing heterogeneous results from different trials, here we make the effort of quantifying the relative contribution of each prognostic factor and, on this basis, develop a management algorithm.

## 2. The Great Divide within Stage II: Low vs. High Risk

In consideration of the heterogeneity of stage II, risk determination and stratification are fundamental to guide therapeutic decisions on adjuvant therapy in these patients. According to international guidelines, stage II colon cancer patients should be stratified into low and high risk of recurrence [4,5,6]. The risk stratification is based on the presence of specific histopathological, clinical, and molecular characteristics. However, there is no standard definition of either group.

Current guidelines recommend adjuvant chemotherapy in the presence of one or more high-risk features without distinguishing the relative prognostic weight among them: pT4, emergency clinical presentation (bowel obstruction or perforation), lympho-vascular and perineural invasion, inadequate lymph node sampling (<12 nodes), and poorly differentiated histology (G3) [4,5,6].

Several subgroup analyses of adjuvant trials have shown that stage II disease with one or more of the abovementioned features has a relative long-term DFS benefit from adjuvant chemotherapy, in terms of hazard ratio (HR), that is similar to that of stage III patients [7,8,9]. This reinforces the need for the identification of the risk of individual patients. In fact, it is very likely that the discrepancies among studies (some showing no benefit of adjuvant chemotherapy, some others showing relative benefits even similar to those obtained in stage III) are due to differences in the baseline risks of the enrolled patient population.

## 3. Prognostic Contribution of Individual Risk Factors

The incidence and the prognostic impact of these risk factors have been extensively investigated within subgroup analyses of large adjuvant trials over the past decades [4]. However, strong evidence supports T stage, lymph node number, grading, and microsatellite status as the main risk factors to be taken into account.

Here, we will determine the relative contribution of each of these factors to the overall risk of relapse of these patients.

### 3.1. T Stage

Pathologic T stage (the grade of penetration in the bowel wall) is one of the most important prognostic factors in colon cancer. The invasion of muscularis propria characterizes pT3, while pT4 represents the involvement of the sierosa, the penetration the visceral peritoneum (T4a), or invasion of adjacent organs (T4b) [10]. The deeper the tumor invasion, the higher the risk of relapse.

According to previous studies, the 5y-OS in stage II with surgery alone ranged between 85–88% for T3 and 70–75% for T4 [11,12,13]. Moreover, a further prognostic difference was observed between T4a and T4b, with 5y-OS of 79.6% and 58.4%, respectively [13].

The 5y-DFS of stage II with surgery alone is 65% and 51% for T3 and T4 respectively [12]. One may be surprised at these very low 5-yr DFS rate for an early stage. Indeed, stage IIB T4N0 has worse outcomes compared to stage IIIA T1–2 N1 colon cancer both with surgery alone and with adjuvant chemotherapy [14]. More specifically, patients with stage IIB colon cancer have a worse 5-year survival (72.2%) than node-positive stage IIIA (83.4%), suggesting that T4 can be an even worse prognostic feature than limited lymph node involvement [11]. The biological rationale of this paradox was hypothesized by the model of Naxerova et al., which distinguished different patterns of metastatic dissemination (distant and lymph node metastases) according to intrinsic aggressiveness of the tumor [15].

With all the general limitations of summarizing different studies’ results, for clinical practice purposes, we can roughly estimate that T4 compared to T3 accounts for a loss of 10–18% in 5y-OS, which makes this factor the most powerful among the others.

### 3.2. Lymph Node Sampling

The proper number of lymph nodes that should be analyzed has been extensively explored for years since few retrieved lymph nodes (LNs) have been associated with poor outcomes.

Swanson et al. have observed that the 5y-OS rates in 35,787 stage II patients differed significantly according to the number of the examined nodes: with 1–7 retrieved LNs, the 5y-OS rate was 49.8%, with 8–12 LNs it was 56.2%, and with > 13 LNs it was 63.4% [16].

The Intergroup 0089 study found a 14% higher absolute 5y OS for stage II patients with > 20 negative lymph nodes examined compared to < 10 negative lymph nodes examined [17]. These results have been confirmed by the Surveillance, Epidemiology, and End Results (SEER) population analyses conducted on 48,446 patients by Gunderson et al. The study showed a 5y-OS benefit of 10% to 20% if 13–15 lymph nodes are removed compared to 0–6 lymph nodes in stage II, with a major survival advantage for T4 cancer [13]. Similar results were observed in other population analyses.

A suboptimal nodal staging may result in a higher proportion of undiagnosed lymph node involvement (stage III disease) and a higher number of patients classified as stage II disease when indeed they are stage III.

This stage migration phenomenon was confirmed by the analysis of the ACCENT Group of more than 18,000 patients enrolled from 1978 to 2002, showing a higher recurrence rate for patients with stage II colon cancer than in earlier studies [18]. Nowadays, all international guidelines recommend collecting and examining at least 10–12 nodes [5,6]. In cases where the lymph node harvest has been particularly poor, oncologists may ask for a pathological reassessment of the sample. If a sufficient number of lymph nodes is not achieved again after this revision, the oncologist should discuss with the patient about the higher risk of recurrence of his condition and consider re-doing surgery if the number of lymph nodes retrieved is particularly low (3–5): certainly, a difficult situation.

With all of the general limitations of summarizing the results of the literature discussed above, we can estimate that an insufficient lymph node sampling is associated with a loss in 5y-OS of about 15%, matching the prognostic weight of T4.

### 3.3. Mismatch Repair Status

The DNA mismatch repair (MMR) system induces apoptosis in damaged cells; its failure leads to an increase in DNA mutations in particular regions of the chromosomes, known as “microsatellite” (short tandem DNA repeats) [19], resulting in the so-called “microsatellite instability” (MSI).

According to the state of the mismatch repair system, it is possible to distinguish two groups of colorectal cancer: MSI-high (or deficiency of the mismatch repair, dMMR) and MSI-low (or proficiency of the mismatch repair, pMMR).

D-MMR is more frequent in early stages (stage II = 15%; stage III = 14%) compared to advanced stages (4%) and in the right side of the colon compared to the left (4 times higher) [19,20].

Untreated patients with stage II dMMR tumors have an improved survival rate (90%) compared to patients with a pMMR status (66%) [20].

Moreover, Sargent et al. showed how the effect of adjuvant chemotherapy in terms of OS changes on the basis of MMR status; more precisely, 5y-OS decreased in dMMR stage II patients treated with fluoropyrimidines compared to those undergoing surgery alone (HR 2.95; 95% CI, 1.02–8.54). These findings indicate that patients with dMMR tumors should not receive fluoropyrimidine-only adjuvant chemotherapy [21].

These findings give the dMMR status both a prognostic and a predictive role in this stage [22,23].

Further studies proved that even the combination with oxaliplatin does not improve the benefit in DFS or OS in these patient subgroups [24].

In summary, dMMR should be considered as a predictor of better survival, with a survival advantage of about 20–25% in 5y OS; pMMR, instead, should not be regarded as a prognostic risk factor.

### 3.4. Grade

A high grade indicates a poorly differentiated disease and is another common feature of poor prognosis [25]. In the SEER analyses on 119,363 patients diagnosed with colon adenocarcinoma (any stage) conducted by O’Connel et al. in 2004, the subgroup with high-grade tumors represents about 20% of the cases [11].

A more frequent incidence of poor tumor differentiation has been observed in stage IIB colon cancer than in stage IIIA: in an analysis conducted by Kim et al., a high-grade tumor was found in twice as many cases of T4N0 than T1–2 N1 [14]. This could explain the different behavior of these two categories of colon cancer.

The International Duration Evaluation of Adjuvant Therapy (IDEA) trial also showed how variable the proportion of high- and low-grade tumors is across studies even conducted at the same time. In fact, within the IDEA trial where four different groups accrued almost 4000 stage II patients, the percentage of high-grade tumors spanned between 11.5% and 57.1% [3].

The analysis by Gill et al. on a population of 3302 stage II and stage III colon cancer patients showed lower 5y DFS and 5y OS in high-grade disease. In particular, high-grade compared to low-grade disease was associated with a loss of 8–9% in 5y DFS in T3N0 and T4N0 tumors (65% vs. 73% and 51% vs. 60%, respectively) [12].

It is hard to summarize the loss in OS accounted for in G3 as compared with G1–2 tumors, given the variability of G3 ascertainment. However, this variable has a lower impact than the factors considered so far and could be estimated in the range of 6–7%.

### 3.5. Clinical Presentation

In the definition of high-risk stage II disease, not only pathological characteristics but also the clinical presentation impacts the prognosis. Bowel perforation or occlusion, indeed, are closely linked to a higher rate of micro-metastases, surgical morbidity, and recurrence [7,26,27].

An emergency colectomy for perforation or obstruction is a stronger prognostic factor than a pathological finding of perforation [28].

However, a weak point of the studies that analyzed these features is the lack of a univocal definition of these conditions (either clinical or pathological). Consequently, the relative risk based on the current literature is hard to assess.

### 3.6. Lymphovascular or Perineural Invasion

The presence of tumor cells in the space surrounding lymphatic or blood vessels defines the pathological feature of lympho-vascular invasion. Similarly, the involvement of nerve fibers depicts the perineural invasion.

In a population of 32,493 patients with stage II colon cancer, lympho-vascular invasion was observed in 11.4% of cases and perineural invasion in 3.8%; double-negative patients comprised about 80%. Their presence is associated with a worse prognosis. In particular, 5y OS decreased by about 6% with lympho-vascular invasion and about 10% with perineural invasion compared to patients without these features [29].

A negative impact on 5y DFS of these factors has also been observed. Indeed, the prospective study by Quah et al. demonstrated a loss of 12% in those patients with lympho-vascular or perineural invasion [30]. In particular, perineural invasion could significantly influence survival outcomes, with worse 5y DFS in stage II perineural-invasion-positive than in perineural-invasion-negative patients (respectively, 29% vs. 82%) [31].

With the current literature, however, it is not possible to assess precisely the survival loss with these features in stage II, and there is no complete consensus about their prognostic value.

## 4. Quantification of Survival Benefit of Adjuvant Chemotherapy: Fluoropyrimidines Alone

We will now examine some of the most relevant studies, trying to estimate the clinical benefit of adjuvant chemotherapy on top of surgery alone, considering the efficacy of fluoropyrimidines alone first and then the addition of oxaliplatin.

The first trial conducted to evaluate the effect of adjuvant fluorouracil in resected colon cancer was the NCCTG 1990, which randomized patients to 12 months fluorouracil + levamisole vs. observation [7]. The advantage of adjuvant chemotherapy was not statistically significant for stage II, probably because the trial was underpowered for this stage. However, the hazard ratio for survival favoring single-agent fluorouracil/leucovorin vs. observation in stage II was substantial and very similar to that observed in stage III.

On the basis of these results, larger trials subsequently compared fluorouracil regimens with surgery alone.

The delta benefit of adjuvant fluorouracil compared to surgery in terms of 5y OS was 2% (82% vs. 80%) in the IMPACT B2 analysis (1999), 5% (81% vs. 76%) in the Intergroup analysis (2004), 4% in the QUASAR trial (2007), and 1% (81% vs. 80%) in the ACCENT pooled analysis (2013) [18,32,33,34].

Notably, a DFS improvement was shown in all studies. The delta benefit in 5y DFS was 4% (76% vs. 72%) in the Intergroup analysis and ACCENT trial, while the IMPACT B2 trial reported an event-free survival (EFS) at 5y of 3% (76% vs. 73%) [18,32,33].

We can summarize that the benefit of fluoropyrimidine alone on top of surgery is 1–5% in terms of 5y OS and 3–4% in terms of 5y DFS/RFS. These results were also observed in the Cochrane meta-analyses conducted by Figueredo et al. in 2008 (DFS benefit of 4–6% and OS benefit of 2–4%) [35].

## 5. Quantification of Survival Benefit of Adjuvant Chemotherapy: Oxaliplatin-Based Doublets

The addition of oxaliplatin to fluorouracil/leucovorin regimens (FOLFOX) in stage II cancer has been explored in two randomized clinical trials [8,36]. In the MOSAIC trial, the combination did not improve the 5y OS (89% vs. 90%) and the 5y DFS (83.2% vs. 80.1%) [37]. NSABP-C07 reported similar results in 5y OS (89.7% vs. 89.6%) and 5y DFS (82.1% vs. 80.1%) [36]. However, the MOSAIC exploratory analysis in high-risk stage II patients highlighted an absolute benefit of 7.7% (81.5% vs. 73.8%) in 5y DFS but no difference in 5y OS was observed (87.6% vs. 87.5%) [37].

The update of MOSAIC at 10y showed no gain in 10y OS in the overall stage II population (78.4% vs. 79.5%) but a difference of 3.7% in high-risk patients (75.4% vs. 71.7%). The 10y-DFS delta was 1.6% in all stage II patients (75.2% vs. 73.6%) and 5.7% in high-risk patients (72.7% vs. 67.0%) [37].

The higher survival results of the MOSAIC trial observed in high-risk patients compared to the overall population constitutes the rational basis for considering the option of adding oxaliplatin in the former subgroup.

We can summarize that in the overall stage II population, the benefit of the addition of oxaliplatin is 2–3% in 5y DFS and 1–2% in 10y DFS, while there is no difference in 5y and 10y OS. These results at 5y were also confirmed by the Cochrane meta-analysis conducted by Figueredo et al. in 2008 (DFS delta of 3.8% and OS delta of 0.1%) [35].

In the high-risk stage II subgroup, the benefit of the addition of oxaliplatin is 7.7% and 5.7% in 5y and 10y DFS; no difference was observed in 5y OS, while a 3.7% benefit was reported at 10y, which raises the point of the relevance of DFS vs. OS in this disease setting.

## 6. Quantification of Survival Benefit of Adjuvant Chemotherapy: 3 vs. 6 Months of Oxaliplatin-Based Doublets

The results of the pooled analysis of the four IDEA studies in 3273 stage II patients showed that 3 months of adjuvant capecitabine plus oxaliplatin (CAPOX) were as beneficial as 6 months (5y DFS of 81.7% vs. 82%), with considerably less toxicity [3]. By contrast, 6 months of FOLFOX yielded better efficacy than 3 months of the same regimen (5y DFS of 79.2% vs. 86.5%), albeit with significantly more toxicity [3].

In summary, in light of the results of IDEA, in high-risk stage II colon cancer 3 months of CAPOX could be an acceptable therapeutic option in the adjuvant setting because the maximum loss of benefit of 3 months of therapy compared to the more toxic 6 months duration is in the range of 1%.

## 7. Quantification of the Survival Benefit of Adjuvant Chemotherapy: The Role of Irinotecan

The role of irinotecan in the adjuvant setting was explored in a few trials with negative results [38,39]. The most relevant study was PETACC-3, a randomized phase III trial that evaluated biweekly infusional fluorouracil/leucovorin alone or in combination with irinotecan as adjuvant chemotherapy for stage II and stage III colon cancer. A subanalysis of 934 patients with stage II colon cancer was reported, but no clinical or pathological characteristics of this population were described. In stage II patients, the addition of irinotecan was not related with a statistically significant survival benefit in 3y and 5y DFS (84.6% vs. 82.5% and 80.9% vs. 76.9%, respectively; *p* = 0.158) or in 5y OS (90% vs. 88.8%; *p* = 0.334) [39].

## 8. Decision Algorithm

**Figure 1:**
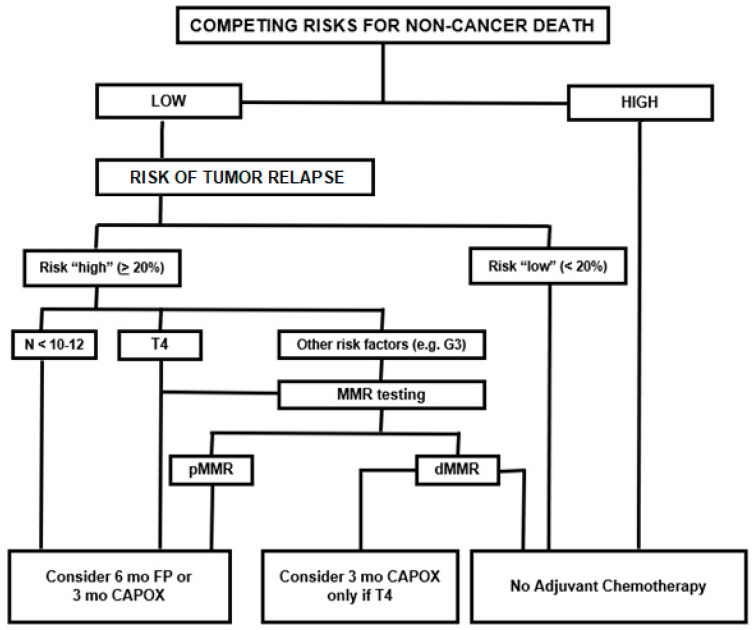
Algorithm for treatment decision in stage II colon cancer. Figure legend: T—tumor, G—grading, N—lymph nodes, mo—months, FP—fluoropyrimidines, CAPOX—capecitabine + oxaliplatin, MMR—mismatch repair, pMMR—proficiency mismatch repair, dMMR—deficiency mismatch repair.

With the purpose of summarizing the abovementioned evidence and translating it for clinical practice, here we propose an algorithm for decision making in stage II colon cancer adjuvant chemotherapy (Figure 1). This has been already partially presented at the ESMO 2019 Congress by Sobrero et al., during a special session dedicated to the management of stage II colon cancer [40].

In routine clinical practice, it is important to assess the clinical conditions of each patient, evaluating the presence of risks for non-cancer death, such as age and comorbidities. This first assessment helps to distinguish patients who are candidates for adjuvant chemotherapy from those who are candidates for follow up only.

In patients with low competing risk, the clinician should estimate the risk of relapse, taking into account the previously mentioned clinical and pathological features.

No one has ever defined what high or low risk is. It is clearly a matter of subjective judgement. However, there is a certain consensus in considering low-risk patients as those with a recurrence risk of < 20%. It is intuitive that with such a small risk, the benefit from adjuvant chemotherapy is supposed to be very low, if any. This group should be approached with observation only.

We can define high-risk patients as those with an incidence of recurrence > 20%. This group can be further divided into two categories from a pragmatic viewpoint. Those patients with T4 tumors or poor lymph node harvest or sampling have the highest risk, in the range of 30–40%. With this size of risk, adjuvant chemotherapy is strongly recommended with 6 months of fluoropyrimidines alone or 3 months of CAPOX. MMR testing could help the decision between these two recommended chemotherapy regimens. Indeed, fluoropyrimidine-only adjuvant chemotherapy should not be given to dMMR patients within this high-risk group.

For stage II patients with other prognostic factors (i.e., G3, vascular invasion, etc.), the MMR status evaluation should be done. Patients with dMMR (about 15% of all stage II colon cancers) do not benefit from adjuvant fluoropyrimidines alone and should only be followed up with no adjuvant treatment. In fact, the dMMR status affords an approximately 20% increase in cure rate on top of the 70% chances of cure by surgery alone, making the recurrence risk really marginal and implying that adjuvant chemotherapy should not be given to this subgroup of patients. All the others should receive 6 months of fluoropyrimidines, for an average benefit of around 2% to 5%.

## 9. Is DFS a Surrogate for OS in Stage II Colon Cancer Patients?

The complexity of the issue of adjuvant chemotherapy in stage II colon cancer regards not only the prognostic weight of each clinical and pathological feature but also the endpoint used to measure the benefit of adjuvant treatment.

In the adjuvant setting of colon cancer, DFS has been studied as a good surrogate endpoint of OS because the recurrence events occur mostly within the first years and are strictly correlated with the subsequent cancer-related death event [41].

A surrogate endpoint has been defined as an alternative end point used to substitute a valid clinical endpoint in order to evaluate earlier the effect of a treatment, with a faster completion of clinical trials, lower costs, and fewer events, allowing more rapid regulatory approval of therapeutic agents. This is of great interest for those cancer types with long post-recurrence survival where a number of active treatment options exist for recurrent disease.

Regarding the role of the DFS surrogacy, a pooled analysis of 18 randomized adjuvant fluorouracil/leucovorin-based chemotherapy trials (ACCENT study) established that 3y DFS is a valid surrogate endpoint for 5y OS in stage III colon cancer [42]. The surrogacy of 3y DFS for 5y OS has been recently evaluated in 8 randomized adjuvant studies conducted from 1998–2009 [43]. With a median follow-up for survival ranging from 5 to 10 years across trials, 3y DFS remains a validated surrogate endpoint for 5y OS in adjuvant trials in colon cancer patients [43].

The same pivotal study on stage III disease did not prove the surrogacy of 3y DFS for 5y OS in clinical trials with only a stage II population, with a hazard ratio for DFS and OS of 0.7 [44]. A possible explanation for this discrepancy is that in contrast to stage III disease, stage II colon cancer patients have a higher likelihood of experiencing lower rates of and longer-term recurrences and higher rates of death without recurrence [45]. Moreover, stage II patients have longer survival following recurrence, so that an extended follow-up is needed to observe a statistically robust association between treatment effects on DFS and OS [45]. For these reasons, OS should be still considered the primary endpoint for stage-II–specific trials [44].

That said, however, we must not forget the intrinsic relevance of DFS from a patient perspective. In fact, DFS is an additional reasonable endpoint of adjuvant therapy because anybody would prefer living longer without disease recurrence and then having a shorter post-progression survival, rather than the opposite. This would lead to the conclusion that DFS could also be the primary endpoint of adjuvant studies in stage II colon cancer, be it a surrogate of long-term survival or not. As a matter of fact, the lack of surrogacy of DFS for OS in stage II as opposed to stage III is counterintuitive and can only be explained by a different biology backing the stage II disease compared to the biology of stage III [15].

## 10. Conclusions

For clinical oncologists, adjuvant chemotherapy still represents a dilemma in stage II colon cancer patients after complete surgical resection. Past clinical trials were inconclusive regarding the efficacy of adjuvant chemotherapy in this setting as no survival benefit was observed in unselected stage II patients. However, high-risk features were identified and are currently used in clinical practice with the purpose of identifying the subgroups most likely to benefit from adjuvant chemotherapy. Among them, the most relevant negative prognostic factors are T4 and insufficient lymph node sampling; each of them accounts for a loss of about 15% in 5y OS on top of the 15–20% risk of relapse of the low-risk patients. Other minor prognostic factors are G3 and the presence of lympho-vascular or perineural invasion, which are associated with a loss of about 6–10% in 5y OS on top of the 15–20% risk of relapse of the low-risk patients. On the other hand, the most relevant good prognostic factor is dMMR, which is linked to a survival benefit of about 20–25% in 5y OS among a mixed population of stage II patients with high-risk features, whose 5y OS would otherwise be in the range of 70%.

The evaluation of available data does not give enough support regarding the prognostic weight of the association of two or more prognostic factors compared to the assessment of every single factor.

We propose here an algorithm where T4 and insufficient lymph node sampling are negative prognostic factors strong enough to, by themselves, recommend the use of adjuvant chemotherapy, while in the presence of other high-risk features the evaluation of MMR should guide the treatment decision.

Results on recent prospective translational clinical trials are awaited to integrate genomic and molecular-based prognostic factors with currently used clinical and pathological prognostic factors in a search for an even more tailored prognostication of stage II colon cancer and the prediction of drug efficacy.

## Figures and Tables

**Table 1:** American Joint Committee on Cancer (AJCC) TNM (Tumor, Nodes, Metastasis) system and staging 8th edition of colorectal cancer.

**T Category**	**T Criteria**
Tx	Primary tumor cannot be assessed
T0	No evidence of primary tumor
Tis	Carcinoma in situ
T1	Tumor invasion of the sub-mucosa
T2	Tumor invasion of the muscuralis propria
T3	Tumor invasion through the muscularis propria into pericolorectal tissues
T4	Tumor invasion of the visceral peritoneum or adherences to adjacent organ or structure
T4a	Tumor invades through the visceral peritoneum including gross perforation of the bowel through tumor and continuous invasion of tumor through areas of inflammation to the surface of the visceral peritoneum
T4b	Tumor directly invades or adheres to adjacent organs or structures
**N category**	**N criteria**
Nx	Regional lymph nodes cannot be assessed
N0	No regional lymph nodes metastases
N1	One to three lymph nodes are positive (tumor in lymph nodes measuring ≥ 0.2 mm), or any number of tumor deposits are present and all identifiable lymph nodes are negative
N1a	One regional lymph node is positive
N1b	Two or three lymph nodes are positive
N1c	No regional lymph nodes are positive, but there are tumor deposits in the subserosa, or mesentery or non-peritonealized pericolic or perirectal/mesorectal tissues
N2	Four or more regional lymph nodes are positive
N2a	Four to six regional lymph nodes are positive
N2b	Seven or more regional lymph nodes are positive
**M category**	**M criteria**
M0	No distant metastasis by imaging, no evidence of tumor in distant sites or organs
M1	Metastasis to one or more distant sites or organs or peritoneal metastasis is identified
M1a	Metastasis confined to 1 organ or site without peritoneal metastasis
M1b	Metastasis to 2 or more sites or organs is identified without peritoneal metastasis
M1c	Metastasis to the peritoneal surface is identified alone or with other site or organ metastases
**Stage**	**Stage criteria**
Stage 0	Tis N0 M0
Stage I	T1–2 N0 M0
Stage II	T3–4 N0 M0
Stage III	AnyT N1–2 M0
Stage IV	AnyT AnyN M1
